# Neighbourhood deprivation across eight decades and late-life cognitive function in the Lothian Birth Cohort 1936: a life-course study

**DOI:** 10.1093/ageing/afad056

**Published:** 2023-04-23

**Authors:** Gergő Baranyi, Federica Conte, Ian J Deary, Niamh Shortt, Catharine Ward Thompson, Simon R Cox, Jamie Pearce

**Affiliations:** Centre for Research on Environment, Society and Health, Institute of Geography, School of GeoSciences, The University of Edinburgh, Edinburgh, UK; Department of Psychology, University of Milano-Bicocca, Milan, Italy; Lothian Birth Cohorts, Department of Psychology, The University of Edinburgh, Edinburgh, UK; Centre for Research on Environment, Society and Health, Institute of Geography, School of GeoSciences, The University of Edinburgh, Edinburgh, UK; OPENspace Research Centre, Edinburgh College of Art, The University of Edinburgh, Edinburgh, UK; Lothian Birth Cohorts, Department of Psychology, The University of Edinburgh, Edinburgh, UK; Centre for Research on Environment, Society and Health, Institute of Geography, School of GeoSciences, The University of Edinburgh, Edinburgh, UK

**Keywords:** cognitive ageing, neighbourhood, social determinants of health, life course, structural equation modelling, older people

## Abstract

**Introduction:**

although neighbourhood may predict late-life cognitive function, studies mostly rely on measurements at a single time point, with few investigations applying a life-course approach. Furthermore, it is unclear whether the associations between neighbourhood and cognitive test scores relate to specific cognitive domains or general ability. This study explored how neighbourhood deprivation across eight decades contributed to late-life cognitive function.

**Methods:**

data were drawn from the Lothian Birth Cohort 1936 (*n* = 1,091) with cognitive function measured through 10 tests at ages 70, 73, 76, 79 and 82. Participants’ residential history was gathered with ‘lifegrid’ questionnaires and linked to neighbourhood deprivation in childhood, young adulthood and mid-to-late adulthood. Associations were tested with latent growth curve models for levels and slopes of general (*g*) and domain-specific abilities (visuospatial ability, memory and processing speed), and life-course associations were explored with path analysis.

**Results:**

higher mid-to-late adulthood neighbourhood deprivation was associated with lower age 70 levels (*β* = −0.113, 95% confidence intervals [CI]: −0.205, −0.021) and faster decline of *g* over 12 years (*β* = −0.160, 95%CI: −0.290, −0.031). Initially apparent findings with domain-specific cognitive functions (e.g. processing speed) were due to their shared variance with *g*. Path analyses suggested that childhood neighbourhood disadvantage is indirectly linked to late-life cognitive function through lower education and selective residential mobility.

**Conclusions:**

to our knowledge, we provide the most comprehensive assessment of the life-course neighbourhood deprivation and cognitive ageing relationship. Living in advantaged areas in mid-to-late adulthood may directly contribute to better cognitive function and slower decline, whereas an advantaged childhood neighbourhood likely affects functioning through cognitive reserves.

## Key Points

- Neighbourhood may contribute to late-life cognitive function, but the life-course association is not yet fully understood.
- Links between life-course area deprivation and general (*g*) and domain-specific cognitive abilities of older adults were explored.
- Living in disadvantaged areas in mid-to-late adulthood was linked to lower levels and faster decline in *g*.
- Associations with domain-specific cognitive abilities (i.e. processing speed) were due to their shared variance with *g*.
- Growing up in deprived areas indirectly contributed to late-life cognition through lower education and restricted residential mobility.

## Introduction

Lifespan changes in cognitive functioning have been observed in non-clinical individuals, with fluid abilities, such as reasoning, memory and processing speed peaking in early adulthood, and declining throughout the second part of life. Crystallised abilities, relying on previously-acquired knowledge and skills, tend to decline well after the 60s [1–3]. Once cognitive deterioration reaches a threshold below which impairments seriously affect everyday functioning, the diagnosis of dementia becomes imminent [2]. Cognitive ageing is, therefore, much more prevalent than dementia, it affects everyday functioning, quality of life and independent living, and it can herald dementia, illness and death [4–6]. Identifying and targeting modifiable risk factors of age-related cognitive changes may slow decline, reduce personal and societal burden, and contribute to healthy ageing.

Social inequalities are persistent in cognitive function and dementia [7–11]. Approximately 20% of dementia deaths are attributable to socioeconomic deprivation with disparities steadily increasing in recent years [12]. In addition to individual-level socioeconomic position, evidence suggests that living in disadvantaged neighbourhoods may independently contribute to dementia and late-life cognitive function [13–16]. However, reviews consistently report the lack of repeatedly-measured area-level exposures [14–16], restricting understanding of when in the life course place-based factors might shape health [15]. The life-course approach helps to understand long-term associations but few investigations assessed sensitive periods in the context of the environment and cognitive functioning [17–19] or dementia [10] and, to our knowledge, none has focussed on life-course neighbourhood disadvantage and age-related cognitive changes. Moreover, studies usually investigate direct associations after confounder adjustment [15]; neighbourhoods, especially during the first half of life, may indirectly contribute to late-life cognition via socioeconomic status.

Existing literature is further limited by brief and relatively insensitive cognitive measurements (e.g. Mini-mental state examination (MMSE)) [15, 16], whereas comprehensive assessments across multiple domains of functioning are rare, especially in a longitudinal setting. Also, cognitive domains are positively intercorrelated, suggesting an underlying general ability factor (*g*) that accounts for much of the variation between cognitive tests [2] and their change over time [20]. Overcoming these concerns requires robust measurements of cognitive domains utilising multiple repeatedly administered tests, and a greater understanding of whether associations are domain-specific, or pervasive across domains linking findings to *g*.

This study explored the relationship between neighbourhood deprivation across eight decades and late-life cognitive function. First, we quantified associations between life-course neighbourhood deprivation and the levels of *g* and domain-specific cognitive function (visuospatial ability, memory and processing speed) at age 70, and their trajectories between age 70 and 82. Second, we tested whether there were unique associations with specific cognitive domains, by separating the cognitive tests’ shared variance from residual variance attributable to single domains. Last, we explored life-course pathways between area- and individual-level socioeconomic status, and cognitive function.

## Methods

### Study participants

We obtained data from the Lothian Birth Cohort 1936 (LBC1936), a longitudinal study of relatively healthy older Scottish adults [21]. The sample was recruited to re-examine some participants of the Scottish Mental Survey 1947 (SMS1947), a nationwide school-based cognitive ability test of 1936-born Scottish schoolchildren, carried out on 4 June 1947 [21]. Surviving participants of SMS1947 living in the Lothian region of Scotland (including Edinburgh) were retraced and contacted [22]. The baseline wave took place between 2004 and 2007, and included 1,091 individuals with an average age of 70 years. Since then, participants have been re-examined at age 73 (2007–10; *n* = 866), 76 (2011–13; *n* = 697), 79 (2014–17; *n* = 550) and 82 (2017–20; *n* = 431) [22].

### Life-course neighbourhood social deprivation

In 2014, a ‘lifegrid’ questionnaire was administered to surviving LBC1936 participants, collecting information on residential history for each decade from birth to the date of completion [22]. The ‘lifegrid’ technique is a valid and accurate way of collecting retrospective residential history [23]. Recall was assisted by ‘flashbulb’ memory prompts (e.g. 9/11 attacks in New York), and by giving participants the option to write down key personal events [17]. Out of 704 contacted participants, 593 provided usable life grid data; addresses were geocoded with automatic geocoders and historical building databases [17, 24].

Decade-specific neighbourhood social deprivation (NSD) scores were constructed for the city of Edinburgh (see details elsewhere) [24]. Briefly, 1941, 1951, 1961 and 1971 NSD was captured using a historical index of multiple deprivations (i.e. population density, overcrowding, infant mortality, tenure and amenities) [24]; 1981, 1991, 2001 and 2011 NSD using the Carstairs index of deprivation (i.e. male unemployment, overcrowding, car ownership and social class) [25]. Data were derived from historical records, and aggregated into a common spatial resolution (1961 census wards; *n* = 23) to support missing data calculation [24]. Decade-specific NSD values were transformed into *z*-scores to ensure comparability [24].

We linked NSD scores to participants’ residential history using time bands of 10 years (e.g. 1941 score to 1936–45 addresses). There was a very high correlation between individual scores closer in time; therefore, we computed average exposure in childhood (1936–55; age 0–19), young adulthood (1956–75; age 20–39) and mid-to-late adulthood (1976–2014; age 40–78) (Supplementary Figure 1).

### Cognitive abilities

We utilised 10 cognitive tests, administered individually across all five study waves at the same location, using the same instruments and the same instructions [21]. Following previous work on their correlational structure [26], tests were grouped into three domains:

*Visuospatial ability* was captured with the Block Design and Matrix Reasoning tests from the Wechsler Adult Intelligence Scale, 3rd UK Edition (WAIS-III^UK^) [27] and the Spatial Span test (average of forwards and backwards) from the Wechsler Memory Scale, 3rd UK Edition (WMS-III^UK^) [28].

*Memory* was measured with the Logical Memory and the Verbal Paired Associates tests from WMS-III^UK^ (total score of immediate and delayed, in each case), and with the Backward Digit Span test from WAIS-III^UK^.

*Processing speed* included the Digit Symbol Substitution and the Symbol Search tests from the WAIS-III^UK^ and two experimental tasks: Four-Choice Reaction Time [29] and Inspection Time [30]. Detailed information on these tests can be found elsewhere [21].

### Covariates

Three sets of covariates were considered (Supplementary Figure 2). The first included age, sex, parental occupational social class (OSC) (professional-managerial [I/II], skilled, partly skilled and unskilled [III/IV/V]) [31] and apolipoprotein E (*APOE*) ε4 allele status (ε4 carriers, not ε4 carriers): confounders for all NSD-cognitive function associations. The second set included childhood IQ (measured as part of the SMS1947 at age 11 with the Moray House test No.12) [21], years spent in (full-time) education and adult OSC (I/II, III/IV/V) [31]: covariates that might mediate the impact of earlier exposures, and confound later ones. The final set of covariates comprised health-related conditions measured at LBC1936 baseline (age 70): smoking status (current smoker, ex-smoker and never smoked), body mass index (BMI) and history (yes and no) of self-reported medical diagnoses (cardiovascular disease; diabetes; hypertension; stroke). As they were concurrently measured with the last NSD epoch (age 40–78) and we could not ascertain that they were true confounders, as opposed to being mediators, we only included them in the sensitivity analysis.

### Statistical analysis

**Figure 1:**
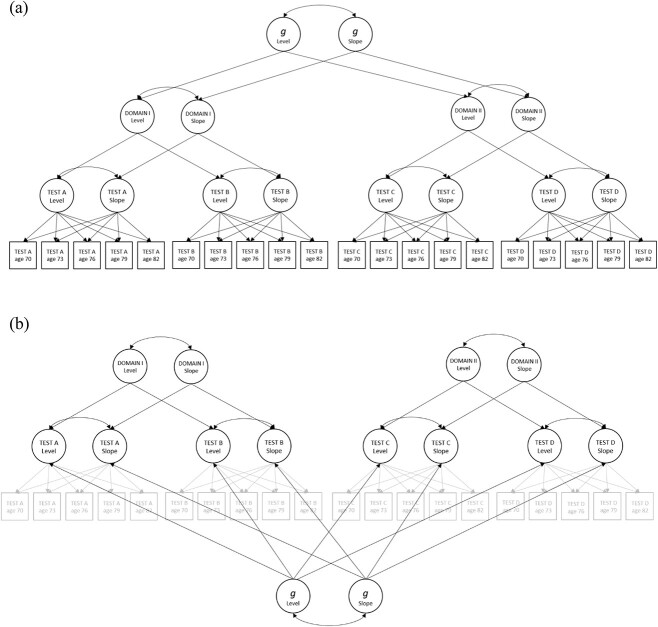
Simplified diagrams of the ‘factor-of-curves’ and the bifactor model of cognitive function. Diagram (a) represents a simplified ‘factor-of-curves’ model. A growth curve for each cognitive test was estimated with latent levels and slopes; loadings on the test slopes were set to 0, 2.98, 6.75, 9.82 and 12.54 to represent the average time (in years) passed between wave 1 and follow-up assessments. Test levels and slopes loaded onto levels and slopes of domain-specific cognitive abilities, which loaded onto levels and slopes of general ability (*g*). Models were run separately for each domain (i.e. visuospatial ability, verbal memory and processing speed) and *g*. Diagram (b) represents a simplified bifactor model. Latent growth curves were constructed for cognitive tests similar to the ‘factor-of-curves’ model. The variance of latent test levels and slopes were partitioned into variance contributing to specific domains and contributing to *g*; the model was run simultaneously for domains and *g*. Time-variant age (i.e. standardised and centred) was included at the measurement level; time-invariant covariates and exposures of interest at the levels and slopes of latent hierarchical domains as regression equations. Variables in squares are measured, variables in circles are latent; double-headed arrows represent correlations.

Data were analysed with latent growth curve modelling within a structural equation modelling framework. The sample was not restricted to individuals with complete data, we fitted models with all available information applying full information maximum likelihood estimation (FIML). We applied the hierarchical ‘factor-of-curves’ approach [32] (see example in Figure 1a) previously used with the LBC1936 [33]. Levels (i.e. intercepts at age 70) and slopes (i.e. trajectories between age 70 and 82) of cognitive test scores were calculated. Linear slopes were modelled by setting the path from the slope to the baseline test score to zero, and using the average time lag between follow-up waves as path weights (i.e. wave 1–2: 2.98 years; wave 1–3: 6.75 years; wave 1–4: 9.82 years; wave 1–5: 12.54 years). Test levels and slopes loaded onto levels and slopes of latent domains (visuospatial ability, processing speed and memory), latent domains loaded onto a higher order *g*, capturing their common variance. Models were fitted with correlations between levels and slopes, for both observed and latent variables. Time-variant age (standardised and centred within wave) was specified at the measurement level; time-invariant covariates and exposures were fitted at the latent domain level as linear regressions. Two nested models were run for each domain separately: Model 1 adjusted for age, sex, parental OSC, *APOE* ε4 allele status; Model 2 additionally for childhood IQ, years spent in education and adult OSC.

Given the intercorrelations between cognitive tests (i.e. *g* accounts for 43% of the variability in test levels and 71% in test slopes in the LBC1936 [34]), we assessed whether NSD-cognitive function associations were domain-specific or pervasive across all cognitive domains. We ran a longitudinal bifactor model (see example in Figure 1b), which partitions the test variance loading onto latent levels and slopes of *g* from those uniquely contributing to specific domains [33]. Before including covariates on the latent variables, we fixed loadings and covariances to values estimated in the measurement model (i.e. model without time-invariant covariates) to ensure model convergence.

We fitted a path model with associations between exposures, outcomes and potential mediators (i.e. childhood IQ, education and adult OSC) modelled in their life-course order, and controlled for Model 1 confounders. Participants’ levels and slopes of *g* were predicted and extracted from the ‘factor-of-curves’ measurement model. Due to temporal overlap in measurement, we specified the link between childhood NSD and childhood IQ as a correlation.

Four sets of analyses assessed the robustness of the ‘factor-of-curves’ models. First, we additionally adjusted for health-related conditions. Second, to capture healthy cognitive ageing (and to reduce the likelihood of recall bias on residential history), we excluded individuals with cognitive impairment, defined as either reporting the diagnosis of dementia or scoring <24 on the MMSE in any of the study waves. Third, as an alternative operationalisation of NSD, we calculated residualised change between epochs (i.e. young adulthood residualised on childhood, mid-to-late adulthood on young adulthood). Last, although estimating latent cognitive trajectories and residuals after covariate adjustment is more optimal in the full sample, we restricted the analytical sample to those who had at least one NSD score.

Effect estimates were reported as standardised coefficients (*β*s) with their 95% confidence intervals (CI). To reduce type 1 errors, we provided false discovery rate (FDR) adjusted *P*-values [35]. Analyses were implemented using the *lavaan* package [36] in R version 4.1.2 [37].

## Results

### Descriptive statistics

Out of 1,091 individuals participating in the baseline wave, 50.23% were male, 27.08% had higher parental OSC (i.e. professional-managerial) and 29.77% were *APOE* ε4 carriers. As residential history was first collected after wave 3, and deprivation scores could only be linked to those residing in Edinburgh, the number of individuals with missing information on NSD was substantial. Individuals with at least one NSD score (*n* = 533) were younger, more likely healthy, had higher IQ and were more likely to belong to higher adult OSC (Table 1). Raw score means and standard deviations of the longitudinal cognitive tests are presented in Supplementary Table 1 for the whole sample; in Supplementary Table 2 for completers (present at all 5 waves) only. Cognitive test scores between ages 70 and 82 declined across all measured tests (reported previously [34]). The distribution of NSD scores and latent cognitive domain values are presented in Supplementary Figures 3 and 4.

**Table 1:** Descriptive statistics

	**Variable**	**LBC1936 sample** ^**a**^ (*n* = 1,091)		**At least one exposure** ^**b**^ (*n* = 533)		*P*-value^c^
		N total	Mean ± SD/N (%)	N total	Mean ± SD/N (%)	
Exposure	Neighbourhood social deprivation					
	Childhood	400	0.387 ± 3.28	400	0.387 ± 3.28	*NA*
	Young adulthood	482	−0.863 ± 2.73	482	−0.863 ± 2.73	*NA*
	Mid-to-late adulthood	494	−2.203 ± 2.82	494	−2.203 ± 2.82	*NA*
Covariates I	Age at baseline (in years)	1,091	69.53 ± 0.83	533	69.36 ± 0.79	^*^^*^^*^
	Sex	1,091		533		ns
	Male		548 (50.23%)		253 (47.47%)	
	Female		543 (49.77%)		280 (52.53%)	
	Parental occupational social class	960		501		ns
	I and II		260 (27.08%)		139 (27.74%)	
	III, IV and V		700 (72.92%)		362 (72.25%)	
	*APOE* ε4 allele status	1,028		507		ns
	ε4 carriers		306 (29.77%)		148 (29.19%)	
	Not ε4 carriers		722 (70.23%)		359 (70.80%)	
Covariates II	Childhood IQ	1,028	100.00 ± 15.00)	506	102.18 ± 14.80	^*^^*^^*^
	Years spent in education	1,091	10.74 ± 1.13)	533	10.81 ± 1.14	ns
	Adult occupational social class	1,070		526		^*^^*^^*^
	I and II		592 (55.33%)		324 (61.60%)	
	III, IV and V		478 (44.67%)		202 (38.40%)	
Covariates III^d^	Smoking status at baseline	1,091		533		^*^^*^^*^
	Current smoker		125 (11.46%)		37 (6.94%)	
	Ex-smoker		465 (42.62%)		235 (44.09%)	
	Never smoked		501 (45.92%)		261 (48.97%)	
	BMI at baseline	1,089	27.78 ± 4.36	532	27.56 ± 4.17	ns
	History of cardiovascular diseases	1,091		533		^*^
	Yes		268 (24.56%)		116 (21.76%)	
	No		823 (75.44%)		417 (78.24%)	
	History of diabetes	1,091		533		ns
	Yes		91 (8.34%)		34 (6.38%)	
	No		1,000 (91.65%)		499 (93.62%)	
	History of hypertension	1,091		533		^*^
	Yes		433 (39.69%)		199 (37.34%)	
	No		658 (60.31%)		334 (62.66%)	
	History of stroke	1,091		533		ns
	Yes		54 (4.94%)		21 (3.94%)	
	No		1,037 (95.05%)		512 (96.06%)	

SD = standard deviation; NA = not applicable; ns = not significant. ^*^P < 0.05; ^*^^*^P < 0.01; ^*^^*^^*^P < 0.001.

^a^Full LBC1936 sample was used for the main analyses.

^b^Subsample including participants with at least one neighbourhood deprivation score.

^c^Difference between participants with and without at least one exposure data was assessed using two-sample *t*-tests for mean differences and on χ^2^ tests for differences in distribution.

^d^Models adjusting for Covariates III are presented in the sensitivity analysis.

### Is life-course neighbourhood social deprivation associated with cognitive function?

**Figure 2:**
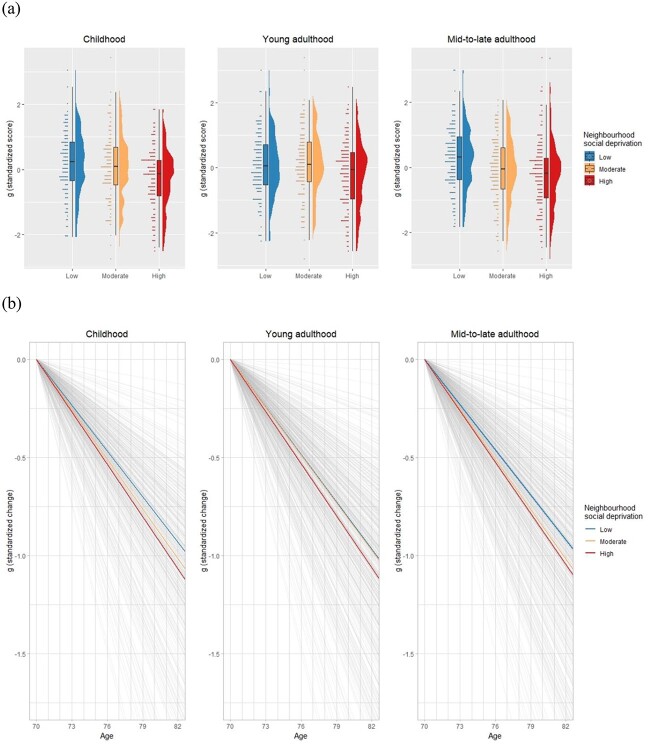
Average (a) intercepts and (b) slopes of general cognitive ability (*g*) by low, moderate, and high levels of neighbourhood social deprivation in childhood, young adulthood and mid-to-late adulthood. Slopes and intercepts of *g* were extracted from the ‘factor-of-curves’ measurement model without adjustment of time-invariant covariates. For this figure, continues measures of neighbourhood social deprivation were split into three equal groups representing low, moderate and high deprivation; intercepts were centred and standardised, slopes were expressed as standardised change (i.e. raw slope values divided by the standard deviation of the raw intercept values). Estimates are based on *n* = 400 for childhood, *n* = 482 for young adulthood and *n* = 494 for mid-to-late adulthood deprivation.

Figure 2 depicts the unadjusted associations between intercepts and slopes of *g* and NSD in three epochs. After adjusting for sex, age, parental OSC and *APOE* ε4 allele status (Model 1), higher mid-to-late adulthood NSD was associated with lower levels of *g*, visuospatial ability, memory and processing speed, and with steeper declines in processing speed (Table 2). On further adjustment for childhood IQ, education and adult OSC (Model 2), the associations between mid-to-late adulthood neighbourhood deprivation and the intercept (*β* = −0.113; 95%CI: −0.205, −0.021) and slope of *g* (*β* = −0.160; 95%CI: −0.290, −0.031), as well as the slope of processing speed (*β* = −0.215; 95%CI: −0.347, −0.083) passed FDR correction (Table 2); the magnitude of the associations was comparable or larger than for indicators of individual socioeconomic status (Supplementary Table 3). Models showed a good fit to the data, with a few exceptions of marginally poorer fits than established cutoffs [38] (Supplementary Table 4).

**Table 2:** ‘Factor-of-curves’ models for the association between neighbourhood social deprivation and cognitive function

Neighbourhood social deprivation		**Model 1**					**Model 2**			
		*β*	95% CI	*P*	*P_FDR_*		*β*	95% CI	*P*	*P_FDR_*
**General cognitive ability (*g*)**										
Intercept	Childhood	-0.126	−0.257, 0.005	0.060	0.120		-0.075	−0.191, 0.041	0.203	0.244
	Young adulthood	0.011	−0.120, 0.143	0.864	0.864		0.086	−0.027, 0.200	0.134	0.244
	Mid-to-late adulthood	**−0.291**	**−0.393, −0.190**	**<0.001**	**<0.001**		**−0.113**	**−0.205, −0.021**	**0.016**	**0.048**
Slope	Childhood	-0.087	−0.240, 0.066	0.263	0.395		-0.104	−0.257, 0.048	0.180	0.244
	Young adulthood	0.025	−0.130, 0.180	0.752	0.864		0.007	−0.147, 0.161	0.929	0.929
	Mid-to-late adulthood	-0.123	−0.250, 0.003	0.056	0.120		**−0.160**	**−0.290, −0.031**	**0.015**	**0.048**
**Visuospatial ability**										
Intercept	Childhood	-0.135	−0.264, −0.006	0.041	0.148		-0.096	−0.209, 0.018	0.099	0.356
	Young adulthood	0.020	−0.108, 0.148	0.761	0.935		0.077	−0.035, 0.188	0.177	0.439
	Mid-to-late adulthood	**−0.251**	**−0.352, −0.150**	**<0.001**	**<0.001**		-0.091	−0.184, 0.001	0.052	0.312
Slope	Childhood	-0.031	−0.250, 0.187	0.779	0.935		-0.048	−0.261, 0.166	0.661	0.936
	Young adulthood	0.001	−0.216, 0.218	0.992	0.995		-0.028	−0.240, 0.184	0.795	0.936
	Mid-to-late adulthood	-0.026	−0.201, 0.149	0.771	0.934		-0.093	−0.270, 0.084	0.305	0.610
**Memory**										
Intercept	Childhood	-0.064	−0.214, 0.086	0.406	0.850		-0.032	−0.168, 0.103	0.640	0.936
	Young adulthood	0.086	−0.063, 0.235	0.259	0.702		0.147	0.015, 0.278	0.029	0.261
	Mid-to-late adulthood	**−0.255**	**−0.372, −0.138**	**<0.001**	**<0.001**		-0.094	−0.204, 0.016	0.093	0.356
Slope	Childhood	-0.041	−0.190, 0.108	0.591	0.935		-0.044	−0.194, 0.105	0.560	0.936
	Young adulthood	0.000	−0.151, 0.152	0.995	0.995		0.008	−0.143, 0.159	0.915	0.936
	Mid-to-late adulthood	0.003	−0.123, 0.130	0.957	0.995		0.018	−0.114, 0.149	0.791	0.936
**Processing speed**										
Intercept	Childhood	-0.054	−0.188, 0.079	0.425	0.850		-0.009	−0.132, 0.114	0.888	0.936
	Young adulthood	-0.037	−0.170, 0.097	0.592	0.935		0.005	−0.117, 0.126	0.936	0.936
	Mid-to-late adulthood	**−0.206**	**−0.312, −0.100**	**<0.001**	**<0.001**		-0.073	−0.174, 0.029	0.160	0.439
Slope	Childhood	-0.088	0.246, 0.070	0.273	0.702		-0.104	−0.262, 0.053	0.195	0.439
	Young adulthood	0.034	−0.125, 0.193	0.676	0.935		0.016	−0.143, 0.174	0.848	0.936
	Mid-to-late adulthood	**−0.186**	**−0.315, −0.057**	**0.005**	**0.023**		**−0.215**	**−0.347, −0.083**	**0.001**	**0.018**

*Note*: Models were run for general ability and for each domain-specific cognitive ability separately. Effect estimates were expressed as standardised betas (*β*). The bold typeface denotes FDR-corrected significance.

Model 1: adjusted for sex, age (time-variant), parental occupational social class and *APOE* ε4 allele status.

Model 2: further adjusted for childhood IQ, years spent in education and adult occupational social class.

### Does general cognitive function account for the associations of domain-specific abilities?

In the longitudinal bifactor model, we removed the test variance of *g* from domain-specific abilities, to test whether domain-specific findings were reflecting cross-domain or unique associations. Models suggested that domain-specific findings were likely due to their shared variance with *g*: only the associations for *g* remained significant in the bifactor model before FDR correction (with similar direction and comparable magnitude as in the main ‘factor-of-curves’ models) and the association between mid-to-late adulthood NSD and processing speed in the fully adjusted model dropped from *β* = −0.215 to *β* = 0.001 (Supplementary Table 5).

### How does neighbourhood deprivation contribute to late-life cognition across the lifespan?

**Figure 3:**
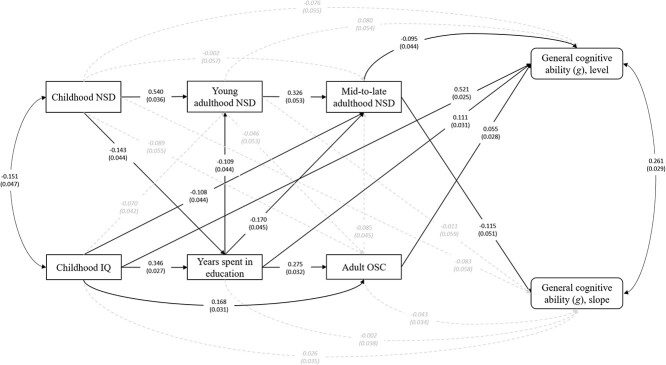
Path diagram depicting life-course associations between neighbourhood social deprivation (NSD), general cognitive ability (*g*) and mediators. Black solid lines represent significant (*P* < 0.05), grey dashed lines non-significant associations; double-headed arrows are correlations. The intercept and slope of *g* were extracted from the ‘factor-of-curves’ measurement model. All presented variables were adjusted for sex, age (time-variant), parental occupational social class (OSC) and *APOE* ε4 allele status (see detailed results in Supplementary Table 6).

We fitted a path model to explore direct and indirect associations between life-course neighbourhood deprivation and late-life cognitive function. After inspecting the correlational structure of variables, we specified all potential pathways. Childhood NSD was significantly correlated with childhood IQ (*β* = −0.151). Although there was no direct effect, higher childhood NSD had downstream associations with *g*. First, it was associated with shorter time spent in education (*β* = −0.143) predicting *g* intercept (*β* = 0.111). Second, it contributed to higher young adulthood NSD (*β* = 0.540), which, in turn, to higher mid-to-late adulthood NSD (*β* = 0.326); and—as shown in the ‘factor-of-curves’ model—mid-to-late adulthood NSD was directly associated with the intercept (*β* = −0.095) and slope of *g* (*β* = −0.115) (Figure 3; Supplementary Table 6).

### Sensitivity analyses

When further adjusting for health-related covariates (i.e. Model 3), the mid-to-late adulthood NSD association was reduced for *g* intercept but remained nominally significant for slopes of *g* and processing speed (Supplementary Table 7). Excluding 56 individuals with cognitive impairment reduced the power, but key findings remained nominally significant (Supplementary Table 8). Operationalising NSD with residualised scores resulted in the same findings as the main models, with additional nominally significant associations for childhood NSD and slopes of *g* and processing sleep (Supplementary Table 9). Finally, restricting the sample to those with at least one NSD score (*n* = 533) suggested a reduced but significant association between mid-to-late adulthood NSD and slopes of *g* (nominally) and processing speed (FDR-corrected) (Supplementary Table 10).

## Discussion

This study examined the relationship between exposure to neighbourhood deprivation across eight decades and the level and slope of late-life cognitive function. There are three key findings. First, living in disadvantaged areas in mid-to-late adulthood was associated with lower *g* at age 70 and its steeper decline between ages 70 and 82. Second, all associations initially identified with apparent domain-specific cognitive abilities were due to their shared variance with *g*. Third, childhood area disadvantage was indirectly associated with *g* through education level and later life neighbourhood deprivation reflecting restricted residential mobility.

Individual socioeconomic conditions across the life course have been associated with cognitive function in older age [8–10, 13], and this study extends the evidence base on the relationship with life-course area-level disadvantage. Living in deprived neighbourhoods during the second part of life was associated with lower levels and steeper declines in *g* and processing speed, with point estimates comparable to or larger in magnitude than for individual socioeconomic position. Associations were 42% larger in magnitude on the slope, as suggested earlier [39]. Advantaged neighbourhoods may differ from disadvantaged ones based on their built and social characteristics. Other research has identified specific neighbourhood characteristics associated with slower cognitive decline [14, 17, 39–43]. Although our findings cannot be linked to specific neighbourhood characteristics that were not part of the analysis, they identify critical periods for sensitivity to neighbourhood disadvantage and motivate future work to examine specific characteristics and their relevance to people of specific age groups. This study emphasises neighbourhood features as key modifiable predictors of ‘successful’ cognitive ageing and the importance of taking a long-term perspective during periods of fiscal restraint and prioritising investment in disadvantaged neighbourhoods.

Partitioning variance between specific and general abilities is a novel contribution to the literature and suggests that associations found between neighbourhood deprivation and specific domains, such as problem-solving [44], semantic memory [45] and processing speed [41] might be an artefact because of their high correlation with *g*. This is particularly pertinent for processing speed; 100% of the association between a decline in processing speed and mid-to-late adulthood NSD was due to shared variance with *g*.

The path model highlighted how life-course individual- and area-level disadvantage jointly contribute to late-life cognitive function. Neighbourhood poverty is associated with lower educational attainment among adolescents [46], which affects late-life cognitive function [13]. Although the current study operationalised the link between childhood NSD and childhood IQ as a correlation, it is plausible that at least part of the causal direction underpinning this association goes from advantaged childhood neighbourhood to better cognitive performance in schools [47]. Childhood environment and subsequent educational enrichment may contribute to ‘cognitive reserves’ through life, which can support the brain’s coping and resilience by delaying pathology and age-related decline [48]. In line with the literature [49], we observed significant ‘tracking by area deprivation’: childhood environment indirectly contributed to cognitive function through selective life-course residential mobility. These pathways are consistent with the ‘chain of risk’ hypothesis [50], whereby one detrimental exposure leads to another one and then to another one, increasing the risk of lower cognitive function and faster decline.

Future research could usefully (i) replicate and refine our findings in larger and more diverse cohorts with higher exposure heterogeneity; (ii) develop a wider range of longitudinal neighbourhood features and examine their contributions to general and domain-specific abilities over the life course; (iii) disentangle how much of the association is related to residential mobility versus urban changes in the neighbourhoods surrounding people and (iv) establish causal pathways between neighbourhood and cognition.

## Strengths and limitations

This study benefitted from a comprehensive set of 10 cognitive tests assessed on five occasions over 12 years, residential history covering eight decades and valid childhood cognitive scores making LBC1936 a unique data source. Longitudinal measurement of cognitive function is essential to understand within-individual changes; although prior test experience might distort longitudinal comparisons up until the age of 65 [3], individual trajectories, especially in older ages are not affected by retest effects. Modelling latent cognitive variables instead of single cognitive test scores provided a more definitive analysis of cognition than previously available, and also reduced the influence of potential measurement error [51]. Exploring the direct and indirect associations in the path model is a further strength of our study.

There are limitations. First, LBC1936 comprises a self-selected, relatively healthy and educated group of individuals. Population-level data suggest that LBC1936 participants had comparatively high childhood IQ [52] and thus a lower risk of mortality [53]. Second, missingness was not random in our sample. However, utilising FIML by fitting models in the context of all available data for confounders and outcomes enabled us to estimate the impact of neighbourhood deprivation more accurately, minimising the bias towards cognitively and physically healthy and higher social class individuals subject to lower attrition/more complete attendance [22]. Third, associations with NSD were calculated based on 400–500 participants living in Edinburgh; we cannot determine whether different effect sizes for NSD epochs were biased by varying sample sizes. Larger samples would be required to detect smaller effect sizes more reliably, especially for early life NSD. Fourth, to provide consistent longitudinal neighbourhood units, we used 1961 census ward geographies, which are unlikely to overlap with participants’ self-defined neighbourhoods, resulting in lower precision and potential misclassification. Fifth, health-related covariates were not available before age 70 leading to unmeasured confounding. Last, mid-to-late adulthood NSD covers almost 40 years, and it is plausible that a more fine-grained temporal analysis would have revealed sensitive periods within this interval; still, we were not able to separate the effects of mid and late adulthood exposure due to their high correlation.

## Conclusions

To our knowledge, we conducted one of the most comprehensive investigations on life-course neighbourhood deprivation and later life cognitive function. Findings highlighted that neighbourhood deprivation in mid-to-late adulthood was associated with lower levels and steeper declines in general cognitive ability. Future studies should identify social and physical neighbourhood features that are pertinent for cognitive ageing differences and establish causal pathways. Given accumulating evidence, neighbourhood context can be considered as policy relevant. Supporting successful cognitive ageing starts in childhood by levelling up the gap in education between neighbourhoods, as well as providing and connecting places where physical, social and mentally stimulating activities could take place.

## Supplementary Material

aa-22-1416-File002_afad056

## Data Availability

The LBCs’ study data have been the subject of many internal (within the University of Edinburgh) and external collaborations, which are encouraged. Those who have interests in outcomes other than cognitive domains are particularly encouraged to collaborate. Both LBC studies have clear data dictionaries, which help researchers to discern whether the variables they wish to use are present; these provide a simple short title for each variable, alongside a longer, common-sense description/provenance of each variable. This information is available on the study website (https://www.ed.ac.uk/lothian-birth-cohorts) alongside comprehensive data grids listing all variables collected throughout both LBC studies and the wave at which they were introduced, an ‘LBC Data Request Form’ and example Data Transfer Agreement. Initially, the Data Request Form is e-mailed to the Lothian Birth Cohorts Director Dr Simon R. Cox for approval (via a panel comprising study co-investigators). Instances, where approved projects require the transfer of data or materials outside the University of Edinburgh, require a formal Data Transfer Agreement or Material Transfer Agreement to be established with the host institution. The process is facilitated by a full-time LBC database manager—there is no charge.
